# Extending the landscape of omics technologies by pathomics

**DOI:** 10.1038/s41540-023-00301-9

**Published:** 2023-08-07

**Authors:** Roman D. Bülow, David L. Hölscher, Ivan G. Costa, Peter Boor

**Affiliations:** 1 Institute of Pathology, RWTH Aachen University Clinic, Aachen, Germany; 2 Institute for Computational Genomics, RWTH Aachen University Clinic, Aachen, Germany; 3 Department of Nephrology and Immunology, RWTH Aachen University Clinic, Aachen, Germany

**Keywords:** Nephrology, Biomarkers, Systems biology

Tissue analysis is vital for investigating disease mechanisms and guiding diagnostics, e.g., in cancer, communicable or non-communicable diseases. During the last decades, technological developments enabled deep molecular characterization of tissue samples. This is particularly driven by omics approaches such as genomics, transcriptomics, proteomics, metabolomics, etc. (Fig. 1)^1^. Omics aims to (quantitatively) analyze possibly all molecules of a specific type in a specimen, the proteome, transcriptome, metabolome, etc. The omics analyses are enabled by specific methods, e.g., genomics by large-throughput DNA sequencing termed Next Generation Sequencing (NGS; Fig. 1). Typically, results from omics analyses contain large numbers of features, e.g., expression of genes, from a large number of instances, e.g., cells. These results allow complex downstream analyses, e.g., uncovering regulatory networks^2^, cell transitions^3^, or key molecular disease drivers^4^. These approaches were missing important information on the spatial organization and structure of the analyzed tissues and organs. Recent approaches allow the integration of the relative position of the investigated instance, most commonly honeycomb-shaped tissue areas, within a given sample using spatial transcriptomics (for example, spatially resolved transcript amplicon readout mapping (STARmap) or NGS barcoding techniques)^5^ or spatial proteomics (e.g., multiplexed antibody-based imaging methods: multi-epitope-ligand cartography (MELC), tissue-based cyclic immunofluorescence (t-CyCIF))^6^. Even though spatial technologies are still evolving, their potential to further extend deep tissue phenotyping is increasingly recognized. The missing piece remains the lack of structural morphological information on an ultrastructural, microscopic, and macroscopic scale. These are the fundamental tasks of pathology, which remains the cornerstone of tissue diagnostics of human diseases.

**Fig. 1:**
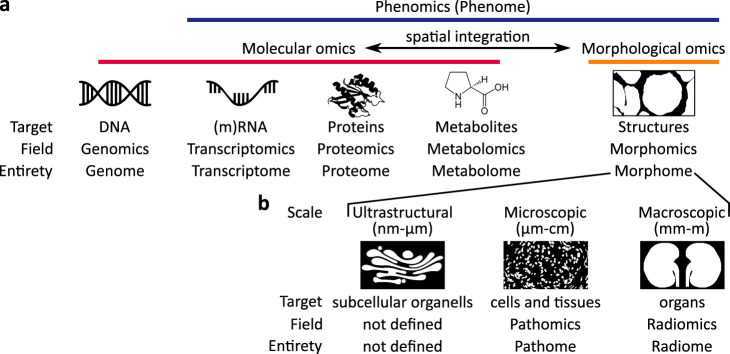
The omics landscape extended by morphology-based approaches. **a** Classical molecular omics can be complemented by morphomics, comprehensively quantifying tissue structure. Particularly, the integration of spatial molecular and morphological omics can provide new insights into tissue organization and diseases. **b** Morphomics analysis at different scales, ultrastructural, microscopic and macroscopic.

The major approach for diagnosing and studying morphological alterations in diseases is using microscopic analysis of histopathology. This analysis changed little since the advent of microscopes and the definition of cellular pathology in the 19th century. It still relies on manual qualitative or semi-quantitative analyses by pathologists, often lacking reproducibility, precision, scalability and throughput. A major breakthrough brings digital pathology, opening the possibilities of automated histopathology analyses and computational pathology^7^. Computational pathology expanded rapidly in recent years, particularly fueled by deep learning technologies. Most approaches utilizing deep learning focus on deriving clinically actionable readouts from histopathology, such as molecular alteration or treatment response prediction, mostly in cancer^8^. While relatively easy to train and use, these approaches have very limited explainability, provide categorical outputs, and are currently not suitable for uncovering novel morphological findings. An alternative approach for data mining of histopathology data uses large-scale, comprehensive extraction of explainable, quantitative features from histological structures identified using semantic segmentation^9^. This approach represents a complementary omics technology for morphology at a microscopic scale, providing multiple features from multiple instances. The instances represent relevant structural units of tissue, e.g., in the kidney, this can be the glomerular, tubular (epithelial), interstitial and vascular compartments or even single cells.

“Pathomics” is the proposed term for this analytical approach^9,10^, “pathome” for the entirety of morphological histology features, and “Next Generation histoMorphometry (NGM)” for the technology, following the terminology of molecular omics (Fig. 1). Pathomics complements a similar approach used in radiology, termed radiomics^11^. Radiomics extracts quantitative features from radiological imaging modalities such as MRI or CT based on structures or volumes of interest, mostly at macroscopic scale^12^. The extracted features are then used to predict clinical outcomes, e.g., disease progression or treatment response. Another discipline tackling morphology is anatomy^13,14^, particularly computational anatomy. This field analyzes and models biological organisms by representing anatomical structures as either 2D- or 3D-shapes to gain insights into structure-function relationships. Computational anatomy is a complementary approach to radiomics, often at a macroscopic scale, but is not restricted to using radiological imaging.

Pathomics and radiomics could be envisaged as part of an overarching “morphomics.” Morphomics might be an ideal term encompassing all morphological descriptors at all scales: ultrastructural (nm-µm), microscopic (µm-cm), or macroscopic (mm-m). The entirety of morphological features would be then called “morphome,” albeit this term is already used in linguistics. The different techniques and approaches used to derive the morphological features at each scale make the subdivision of morphomics into pathomics and radiomics meaningful (Fig. 1).

Another overarching term in omics is phenomics (Fig. 1), which describes the comprehensive analysis of phenotypes characterized by multiple traits. This is typically done by integrating high-throughput data from multiple fields, in principle, all molecular omics apart from genomics, and would also include all morphomics approaches^15^.

Pathomics seamlessly integrates into the multi-omics landscape, providing the missing in-depth analyses of structural changes in tissues on a microscopic level (Fig. 1). In each tissue, microscopic histological structures can be defined on several levels. The currently smallest individual units are subcellular organelles. However, mainly nuclei can be visualized in standard histology, other subcellular organelles, or small organisms like viruses, and analysis of their morphology, normally requires ultrastructural analysis using electron microscopy. The next level is represented by single cells, followed by cells organized in functional units or compartments in association with the extracellular matrix, such as glands, interstitium or stroma, vessels and nerves. All these structures can be observed using light microscopy. Finally, the organization of these compartments defines the structure of the specific organ or tissue. These levels represent a connected multilevel hierarchical organization. A wide range of features can be extracted from histological structures, such as size (area, length of axes, etc.)^16^, shape (circularity, eccentricity, elongation, etc.)^9^, color (hue, intensity, etc.) or texture (homogenous, heterogenous, etc.)^11^. In addition, features can be derived from the relationship of histological structures to each other: distance between similar structures or structure density (for example, cellularity of a gland) and geometric orientation (for example, nuclear major axis orientation)^17^. Lastly, several structures can build a tissue architectural unit that itself can express features, such as structure density or orientation of structures within and between units. Some features, such as color or texture, can be extracted from arbitrary regions of interest, such as image tiles, i.e., without defining a biologically meaningful structure expressing the features. Compared to molecular omics, which often require specific, costly infrastructure and sample preparation, pathomics uses the already available histological slides from the clinical routine and can be run on a rather standard computational infrastructure (Fig. 2).

**Fig. 2:**
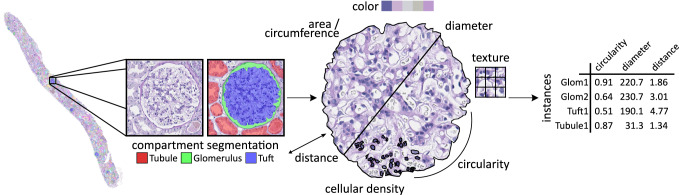
Calculation of common pathomics features within a segmentation framework. Tissue such as kidney tissue can be segmented by deep learning-based algorithms into major histological compartments (e.g., glomeruli, tubules). Based on the segmentation results, various features regarding morphometry (e.g., area, shape, diameter), color or texture are calculated. The output of the framework is a high-dimensional matrix with each instance and its respective features similar to classic molecular omics.

Several key developments are needed to advance pathomics and facilitate its adoption in research and clinical diagnostics. There is a need to agree on standard definitions of histopathological structures and image features to improve comparability between studies, as was initiated for radiomics^12^. Although only very few pathomics studies exist, the terminology differences limit comparability. Histopathology is confronted with large variability, especially regarding the quality and appearance of stains. Normalization methods that can align the appearances of stains from various laboratories without changing the tissue geometry will be essential. It would be desirable to make pathomics datasets publicly available to facilitate their reuse. Ideally, the corresponding images should be publicly available as well since structure definitions might change over time. However, this is often hindered due to patient privacy and legal issues. New initiatives, such as the BigPicture^18^ project, could potentially resolve this problem and offer a repository for digital pathology and pathomics.

Downstream analyses of pathomics data and their integration with other omics, potentially analyzed across different organs, represent an important avenue of future research. Since the location of histopathological structures is inherently traceable (“spatial”), an approach combining pathomics with spatial transcriptomics or proteomics might be an obvious starting point, e.g., using graph neural networks^19^. The integration of pathomics with radiomics, as two “morphomic” approaches tackling different scales, might be possible by selectively correlating corresponding areas within radiological imaging and histopathology. Novel radiologic imaging modalities with high resolution might facilitate this integration^20^. Another integration strategy might be multimodal learning, using different data fusion approaches as recently described for radiomic and pathomic data^21^. Available methods, such as clustering, least absolute shrinkage and selection operator (LASSO)^22^ or recursive feature elimination^23^, might prove useful to identify biologically relevant features. Patient-level aggregation of pathomic information^24^ will be critical to facilitate the integration of pathomics into clinical prediction models.

Morphology-based omics, including pathomics, might offer a missing piece in the study of disease mechanisms across scales.

## Reporting summary

Further information on research design is available in the Nature Research Reporting Summary linked to this article.

### Supplementary information

Reporting Summary
